# Performance Evaluation of Calcined Phosphogypsum Reinforced with Basalt Fiber and Calcium Carbonate Whiskers: A Study on Individual and Mixed Tests

**DOI:** 10.3390/ma17081725

**Published:** 2024-04-10

**Authors:** Yong Jiang, Jichuan Huo, Yonglin Lei, Lujun Jia

**Affiliations:** 1 Basalt Fiber and Composite Key Laboratory of Sichuan Province, Dazhou 635000, China; jiangyong7586@163.com; 2 School of Materials and Chemistry, Southwest University of Science and Technology, Mianyang 621000, China; leiyonglin@163.com; 3 School of Materials and Construction, Mianyang Polytechnic, Mianyang 621000, China; 10425@mypt.edu.cn

**Keywords:** calcined phosphogypsum, basalt fiber, calcium carbonate whisker, strength

## Abstract

In an effort to appropriately address the insufficient mechanical properties of calcined phosphogypsum, this research intends to explore how to utilize basalt fiber and calcium carbonate whiskers as reinforcing agents. The study delves deep into their impacts on the flexural and compressive strength, toughness, water resistance, and tensile strength of calcined phosphogypsum. In the individual tests, basalt fibers with different lengths (3 mm, 6 mm, 9 mm, and 18 mm) were added at dosages of 0%, 0.5%, 1.0%, and 1.5%, respectively. As clearly demonstrated by the research findings, basalt fiber effectively reinforces the flexural and compressive strength, toughness, and tensile strength of calcined phosphogypsum, though compromising water resistance. Among the various fiber lengths, the 6 mm fibers impose the most advantageous influence on the performance of calcined phosphogypsum. Afterwards, a test was conducted to explore how cross−scale fibers affect the properties of calcined phosphogypsum by mixing 6 mm basalt fibers and calcium carbonate whiskers. As illustrated by the experimental findings, calcium carbonate whisker refines the pores, thereby elevating the flexural strength and toughness of calcined phosphogypsum. Furthermore, it compensates for the water resistance limitations associated with the sole utilization of basalt fiber while further augmenting the tensile strength and strain capacity. Nonetheless, it is particularly noteworthy that heightening the dosage of both calcium carbonate whiskers and basalt fibers concurrently gives rise to augmented porosity of phosphogypsum and lowered compressive strength.

## 1. Introduction

As known to all, phosphogypsum (PG) is an industrial by-product predominantly composed of CaSO_4_·2H_2_O, which is produced through the wet-process phosphoric acid process. In general, around 4–6 tons of PG are generated for every ton of phosphoric acid produced [1,2]. Along with CaSO_4_·2H_2_O, PG also contains fluorapatite, goethite, and quartz, as well as small quantities of phosphate, anatase, magnetite, monazite, and quartz. Aside from that, it contains trace amounts of heavy metals such as Cd, Ni, and Cu [3,4,5]. The significant quantity of PG generated can result in water and soil pollution, which makes the effective utilization of PG a highly concerning issue for researchers.

Employing phosphogypsum as a valuable resource represents a significant and advantageous approach for the production of construction materials [1]. Extensive studies has been conducted to explore the use of phosphogypsum instead of natural gypsum in the production of Portland cement, and the feasibility of this approach has been demonstrated through extensive research [6,7,8,9,10]. This substitution is conducive not only to mitigating solid waste issues but also to minimizing the dependence on natural gypsum reserves for cement production. Substantial research efforts have been devoted to the exploration of innovative cementitious materials incorporating phosphogypsum. For instance, Nguyen [11] and Liao [12] successfully synthesized supersulfated cement possessing exceptional mechanical properties utilizing phosphogypsum. Zhang [13] implemented a synergistic combination of slag powder, Portland cement, and phosphogypsum, resulting in a composite cementitious material displaying an amazing compressive strength of 42.6 MPa and a softening coefficient of 89%. Isteri [14] prepared ferrite calcium sulfoaluminate Belite cement from metallurgical waste and phosphogypsum as raw materials. Moreover, the literature documents investigations into the utilization of phosphogypsum as road materials [15,16], unfired ceramsite [17], and unfired bricks [18].

Generally speaking, uncalcined phosphogypsum is non-cementitious in nature. Nevertheless, the calcination process at 160–180 °C transforms phosphogypsum into calcined phosphogypsum predominantly comprising β-CaSO_4_·0.5H_2_O. This thermal treatment technique expands the potential applications of phosphogypsum, which enables it to substitute natural building gypsum in the production of universally employed construction materials such as mortar, blocks, and hollow slats. Nonetheless, the mechanical properties of calcined phosphogypsum are typically less superior than those of natural building plaster [4,19], chiefly owing to the presence of impurities such as phosphate rock, residual acids, metal compounds, and non-degradable organic matter [20,21]. These impurities have been observed to alter the crystal morphology of calcium sulfate dihydrate, resulting in a transition from long interlocking needle-like crystals to prismatic and lath-like crystals with compromised crystal stacking, which thereby gives rise to lowered strength [22]. To elevate the mechanical properties of inorganic cementitious materials, the inclusion of fibers has emerged as a prevalently adopted strategy. Basalt fiber (BF), renowned for its corrosion resistance, high temperature stability, and exceptional tensile strength, has been universally applicable in the realm of building materials [23,24,25].

As already demonstrated by several studies, BFs can exert a positive influence on the mechanical properties [26] and high temperature resistance [27] of gypsum-based materials. When BFs is combined with other fibers, it exhibits an enhanced reinforcing effect. Lv [28] conducted an orthogonal test, incorporating basalt fiber and polyvinyl alcohol fiber into desulfurized gypsum. Just as revealed by the study findings, organically combining these two fibers ameliorated the mechanical properties, water resistance, and durability of the material. It is crucial to note that the maximum absolute dry flexural strength and compressive strength augmented by 70.05% and 64.52%, respectively. Whiskers are one-dimensional nanomaterials with microscopic fibrous structures. As persuasively demonstrated in the study conducted by Jian [29], when modified with calcium carbonate whiskers (CW), the flexural strength of desulfurized gypsum can be heightened by more than 30%. Furthermore, this modification can positively affect the reinforcement of water resistance. Other studies have demonstrated that blending millimeter- and nanometer-scale fibers can intensify the interaction between fibers and the matrix, thereby effectively ameliorating the mechanical properties of cement-based building materials [30,31]. Nonetheless, rare studies have been found on the reinforcement of gypsum through the mixing of cross-scale fibers, necessitating more comprehensive and systematic exploration into its feasibility.

Current research probes deep into the impact of BF and CW on the properties of phosphogypsum through both individual and mixed tests. It is especially innovative to mix BF and CW so as to explore the effects of different scale fibers on the properties of phosphogypsum. The study can not only provide brand new ideas for optimizing the properties of gypsum-based building materials but also lays scientific foundation for facilitating the application of industrial by-product gypsum in the field of building materials.

## 2. Materials and Test Methods

### 2.1. Materials

**Figure 1:**
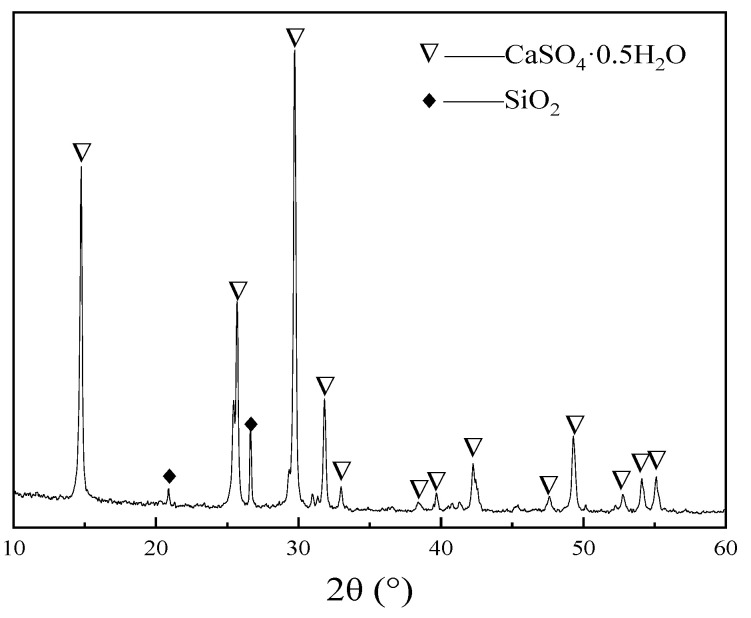
XRD pattern of CPG.

**Figure 2:**
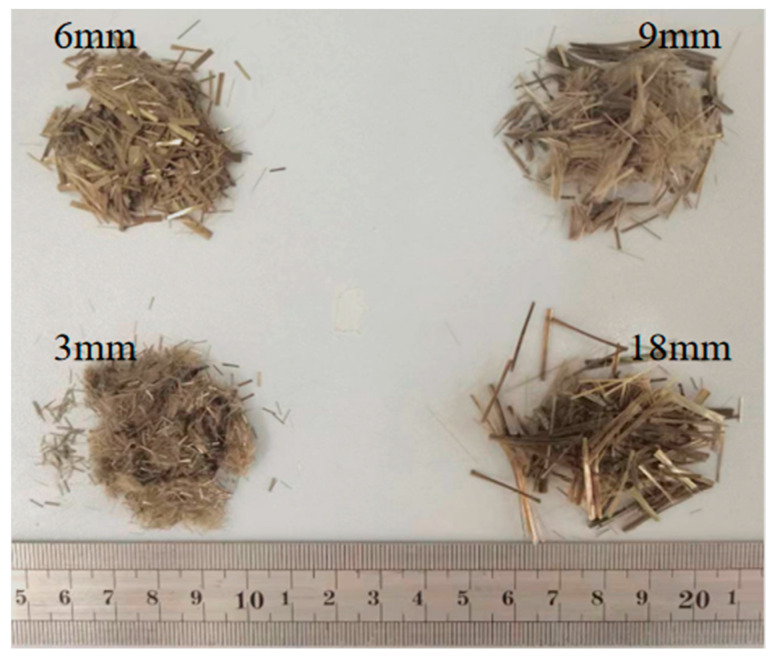
Appearance of different lengths of BFs.

**Figure 3:**
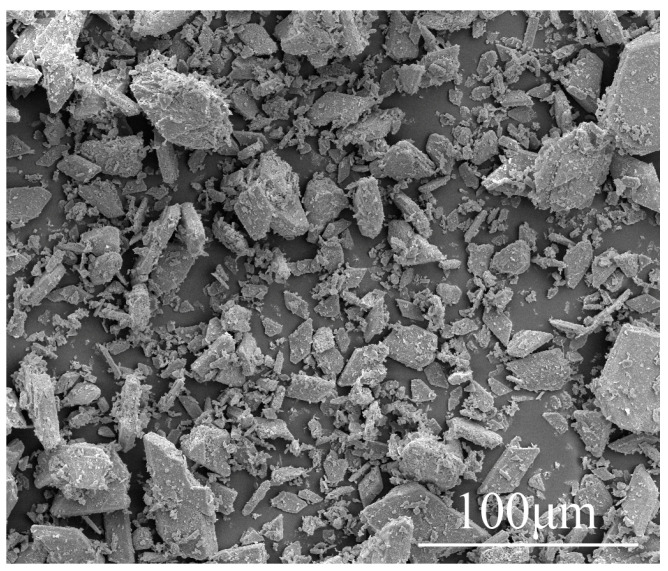
SEM images of CPG.

**Figure 4:**
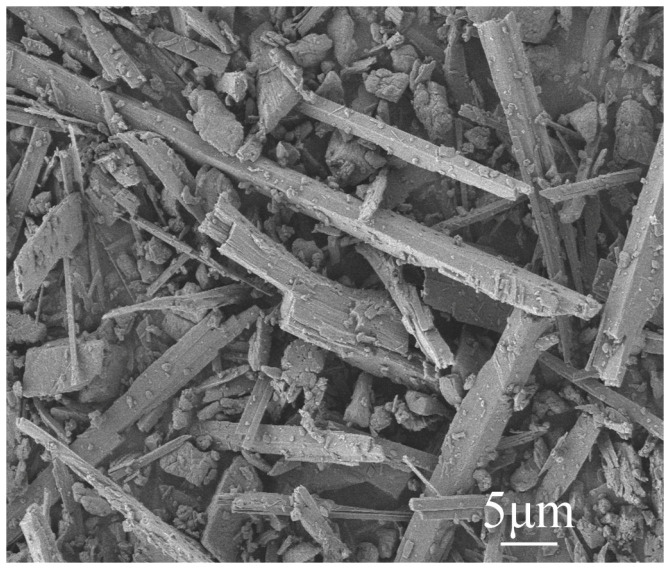
SEM images of CW.

**Figure 5:**
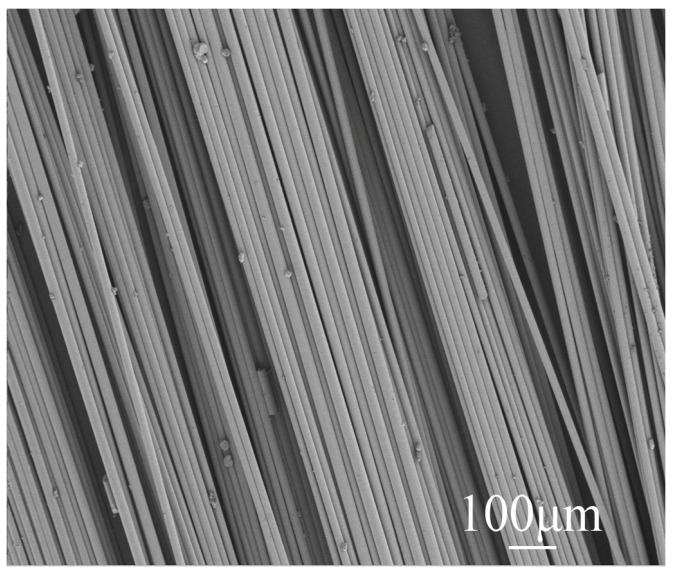
SEM images of BF.

Calcined phosphogypsum (CPG) was provided by Sichuan Longmang Phosphorus Chemical Co., Ltd. (Long mang: Deyang, China), and its XRD analysis results are shown in Figure 1. Analysis shows that the main components of CPG are CaSO_4_·0.5H_2_O and SiO_2_. Protein-based gypsum retarder and CW were provided by Sichuan Tong Xingyuan Building Material Technology Co., Ltd. (Tong Xingyuan: Mianyang, China), and BFs was provided by Sichuan Errun Building Material Co., Ltd. (Ya ’an City, Sichuan Province) Figure 2 displays the appearance and morphology of BFs. Figure 3, Figure 4 and Figure 5 depict the SEM images of CPG, CW, and BF, separately. As apparently revealed in the SEM image, the CPG particles exhibit varying sizes, with diameters less than 50 μm, and they possess edges and corners. The CW particles are chiefly fibrous, interspersed with non-fibrous particles. The fiber diameter measures less than 5 μm, but the length and fineness of the fibers exhibit unevenness, giving rise to an aspect ratio ranging from 10 to 30. The BF fibers display a regular cylindrical shape with a smooth surface. Table 1 exhibits the raw material parameters provided by the vendors.

### 2.2. Experimental Design

Table 2 exhibits the mixing ratios used in the study. The mass ratio of the raw materials comprising the gypsum slurry was CPG-water-wretarder = 1:0.45:0.001. In the individual tests, BFs of 3 mm, 6 mm, 9 mm, and 18 mm were selected, and the BFs’ mixing amount was set to 0, 0.5%, 1.0%, and 1.5% for each length by mass of gypsum. 6 mm BF and CW were selected for the mixed test. Furthermore, the dosages of BF and CW were set at 0.5%, 1.0%, 1.5%, and 0.5%, 1.0%, 2.0%, separately.

### 2.3. Sample Preparation

**Figure 6:**
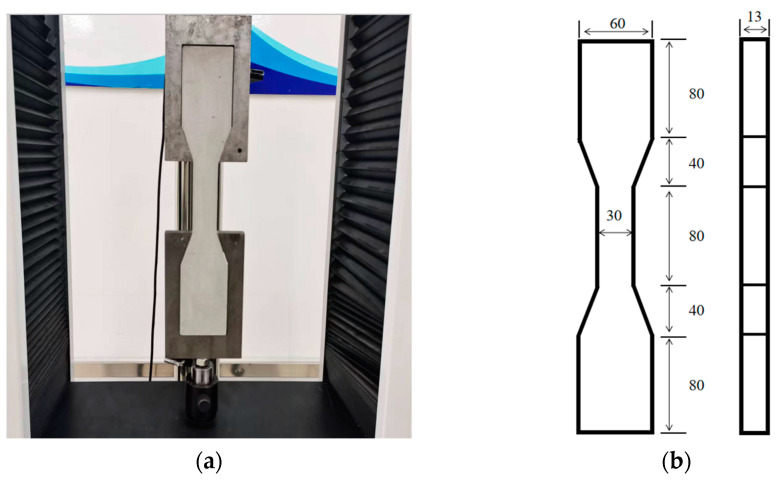
Tensile test method and sample size (**a**): The tensile strength of the sample was tested by a universal testing machine; (**b**): Sample size, mm.

After weighing all the raw materials, CPG, CW, and the retarder should be added to the mixer. Stir the mixture for 20 s and then add water. Continue stirring for 30 s until a uniform slurry is obtained. Afterwards, add BFs to the mixer and stir for an additional 120 s to ensure proper dispersion of the fibers. Once the mixing is complete, pour the slurry into the designated mold. Use a vibration table to vibrate the slurry for 60 s so as to remove air bubbles. Then, place the mold in an environment with a temperature of 20 ± 2 °C and a relative humidity of 90 ± 2% for 24 h to allow for curing. After the curing process, the mold can be removed. For the tests of flexural resistance, compressive resistance, water absorption, and softening coefficient, a sample measuring 40 mm × 40 mm × 160 mm should be used. As depicted in Figure 6, a dumbbell−shaped sample should be used for the tensile strength test.

### 2.4. Test Methods

Flexural and compressive strength: The prepared sample should be placed in a blast drying oven at 40 ± 2 °C until it reaches a constant weight. Subsequently, the flexural and compressive strength should be measured using a 40 mm × 40 mm × 160 mm sample, following the Chinese standard GB/T 9776-2008 [32]. The loading rates for flexural and compressive strengths were 50 N ± 10 N and 2400 N ± 200 N, respectively. Apart from that, the compressive strength should be tested by soaking the dried sample in water for 24 h. The softening coefficient can be calculated by comparing the compressive strength after soaking to the compressive strength of the dried sample. Water absorption can be determined by subtracting the dry weight from the wet weight of the sample and dividing it by the dry weight. Three samples were tested for each set of experiments; then, the mean and error were calculated accordingly.

Tensile strength: The dumbbell-shaped sample was dried and then subjected to a tensile strength test by employing a universal testing machine. The loading rate used was 0.3 mm/min. During the test, stress and strain data were automatically collected by a computer. As a consequence of the discreteness existing in the test values for tensile strength, five samples were tested in each group; data exceeding 15% of the mean value were deleted, and the mean and error values of the remaining data were calculated.

Microscopic analysis: In an effort to investigate the dispersion state of the fibers in the gypsum matrix, SEM analysis was performed to delve into the hardened samples by employing Zeiss Gemini 300. (Zeiss Gemini 300: Jena, Germany, Carl Zeiss Corp) Apart from that, samples underwent mercury intrusion porosimetry (MIP) analysis using AutoPore Iv 9510 (AutoPore Iv 9510: Norcross, GA, USA, Micromeritics Instrument corp) to gather information on pore volume, porosity, and other relevant parameters.

## 3. Test Results and Analysis

### 3.1. Individual Tests

#### 3.1.1. Flexural Strength

**Figure 7:**
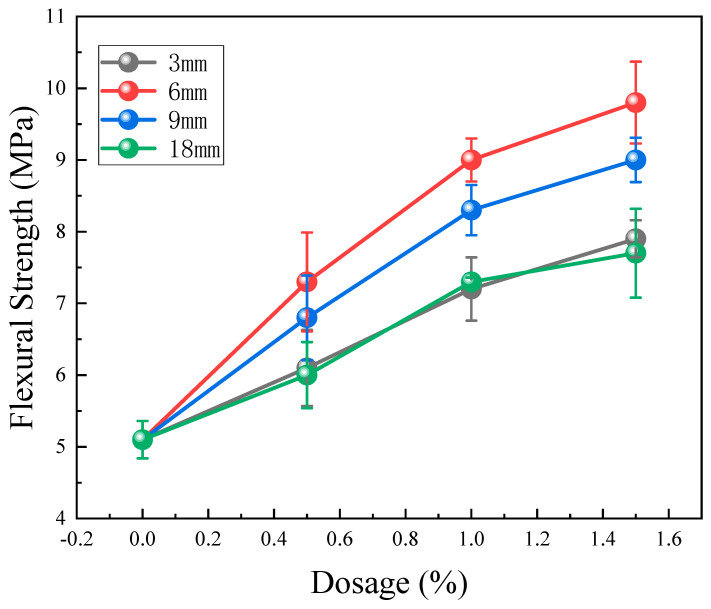
Test results of flexural strength.

The test results of the flexural strength test are presented in Figure 7. Dissimilar lengths of BFs impose a positive influence on the flexural strength of CPG. As the BF content increases steadily, the flexural strength displays an upward trend accordingly. The augmentation is more conspicuous when the BF content ranges from 0% to 1.0%. Nevertheless, the upward trend slows down when the BF content exceeds 1.0%. Among the BFs’ lengths, 6 mm and 9 mm are the most effective sizes in increasing the flexural strength, followed by 18 mm and 3 mm. The flexural strength reaches its highest peak of 9.8 MPa when the 6 mm BF content is 1.5%, which is 92.2% higher than the blank group without BF.

BF presents exceptional tensile strength and fracture toughness, displaying a strengthened mechanical bite force and grip force as fiber length and content scale up. This intensified interfacial interaction effectively resists internal tensile stress within the matrix, triggering prominent energy absorption and lessened internal damage and cracking [33]. In the three-point flexural test, the upper region is subjected to compression, while the lower region undergoes tension. Shorter 3 mm fibers are susceptible to pull-out during the tension phase, with slipping and debonding mechanisms displaying trivial influence on energy dissipation. As a consequence, the 3 mm basalt fibers make a trivial difference to flexural strength. Although 18 mm fibers are long enough, it is noteworthy that longer fibers encounter difficulties in spreading and achieving uniform dispersion during the stirring process, giving rise to agglomeration and defect formation [34]. In comparison, BFs measuring 6 mm and 9 mm are determined to be more efficient in reinforcing flexural strength.

#### 3.1.2. Compressive Strength

**Figure 8:**
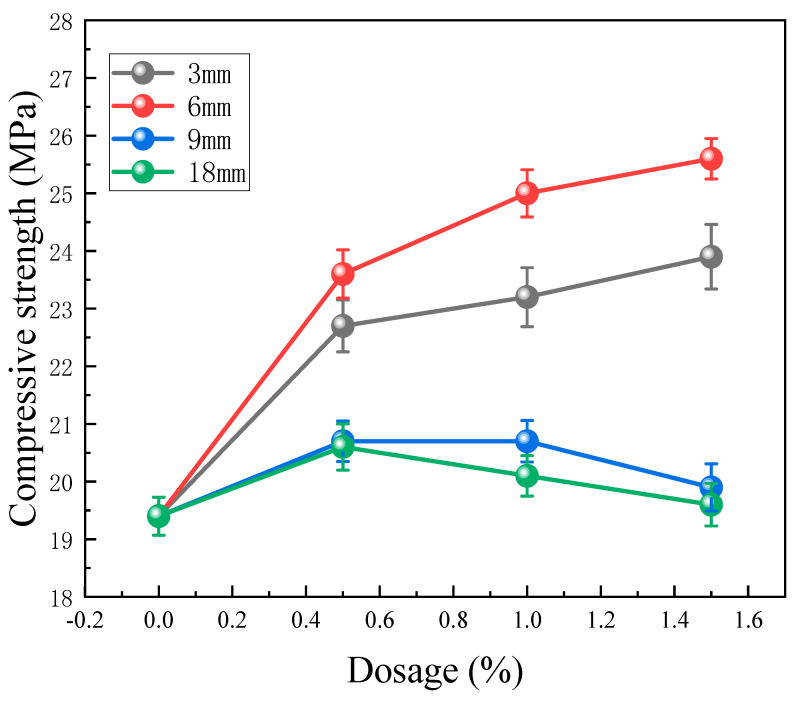
Test results of compressive strength.

As displayed by the compressive strength test results depicted in Figure 8, the addition of BFs augments the compressive strength of CPG. To be specific, an incremental improvement in compressive strength is evident with the increasing content of 3 mm and 6 mm BFs. Nonetheless, the impact of 9 mm and 18 mm BFs on elevating compressive strength is not as remarkable as expected, displaying an initial augmentation followed by a reduction when their contents heighten continuously. What is critical to mention is that when the content of 6 mm BF reaches 1.5%, the compressive strength reaches its maximum value at 25.6 MPa, presenting a significant 32.0% increase in comparison with the control group. Such betterment can be ascribed to the rigidity imparted by BF, which fortifies the matrix and impedes crack expansion during compression, thus boosting the compressive strength of CPG. Notably, the shorter 3 mm and 6 mm BFs are advantageous for the amelioration of compressive strength, as their random dispersion inherently lessens unfavorable stress distribution [34]. Conversely, the longer 9 mm and 18 mm BFs are prone to agglomeration defects within the matrix [35], which brings about less pronounced reinforcements in compressive strength.

#### 3.1.3. Toughness

**Figure 9:**
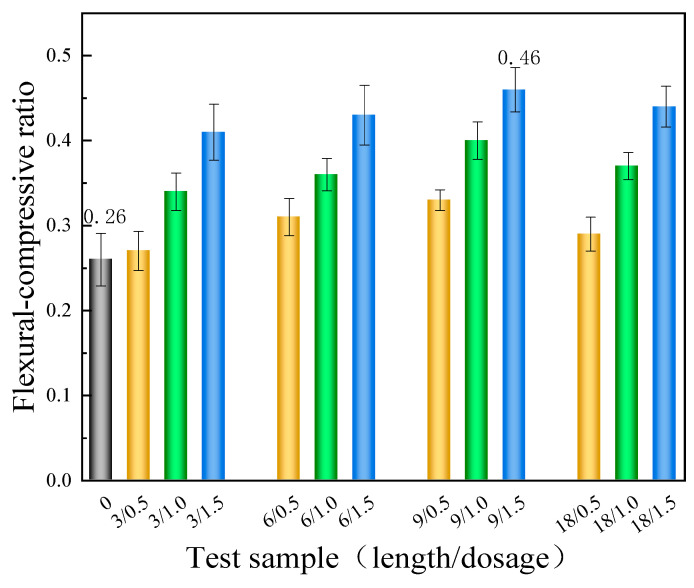
The flexural–compressive ratio of the samples (3/0.5 represents the test group with a 3 mm BF content of 0.5%, and others are similar).

The toughness of gypsum is commonly evaluated using its flexural–compressive ratio. A higher ratio indicates better toughness of gypsum. As clearly depicted in Figure 9, the ratio is determined by calculating the flexural and compressive strengths. The blank CPG has a ratio of only 0.26. Nevertheless, when BFs with varying lengths and dosages are added, the ratio significantly increases as a whole. Apart from that, for the test group mixed with BFs of the same length, the ratio gradually increases with the augment in BF content. Notably, the test group mixed with 9 mm BF exhibits the highest flexural–compressive ratio. At a dosage of 1.5%, the ratio reaches 0.46, which is 76.9% higher than that of the control group.

#### 3.1.4. Water Absorption and Softening Coefficient

**Figure 10:**
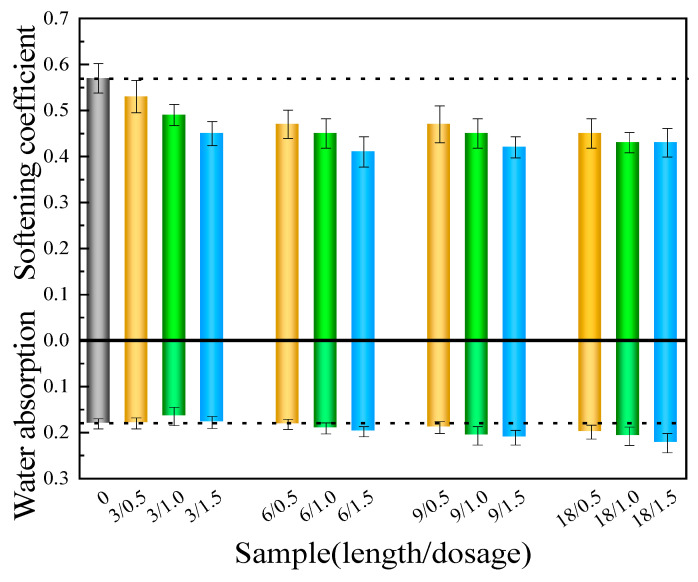
Test results of water absorption and softening coefficient.

Figure 10 displays the results of the water absorption and softening coefficient assessments. As compared with the control group, the experimental groups, where the matrix is mixed with BFs of different lengths and quantities, demonstrated decreased softening coefficients. Furthermore, this experiment uncovered an inverse correlation between the softening coefficient and the content of BFs. In particular, the test group containing 3 mm BF exhibited a marginally lower water absorption rate than the control group, while the remaining groups displayed higher rates of water absorption. This dissimilarity can be ascribed to the presence of a notable quantity of monofilament fibers within the 3 mm staple fibers. These fibers are intricately dispersed throughout the matrix and interfere with pore connectivity, thus contributing to diminished water absorption [36]. Nonetheless, the hydrophilic nature of the basalt fiber surface, combined with its smooth characteristics, hinders strong bonding with the matrix, which brings about a weak interface that promotes the dissolution of hydration products and facilitates the free movement of water molecules [37]. As a consequence, if mixed with BFs of other lengths, the water absorption trend of the experimental group can be elevated correspondingly. On this basis, the inclusion of BFs does not elevate the water resistance of CPG.

#### 3.1.5. Tensile Strength

**Figure 11:**
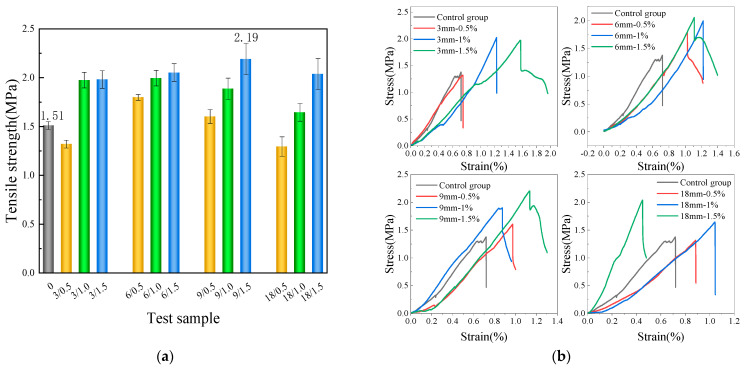
Tensile strength test results (**a**): Average tensile strength; (**b**): typical stress–strain curve.

Figure 11a depicts the test results of tensile strength. As apparently demonstrated by the findings, except for the 3/0.5 and 18/0.5 groups, the strength of the remaining groups has increased accordingly. The decrease in strength in the 3/0.5 group can be attributed to the occurrence of harmful stress concentration when shorter BFs are mixed into the CPG at a lower dosage [37]. The decrease in strength in the 18/0.5 group may be due to the uneven dispersion of fibers. When BFs of the same length are added, the tensile strength increases with higher BF content. Nevertheless, when maintaining the same BF content, the tensile strength first increases and then decreases with the increment in BF length. As a whole, BFs with lengths of 6 mm and 9 mm are more effective in reinforcing tensile strength in comparison with those with with lengths of 3 mm and 18 mm. The 9/1.5 group exhibits the highest tensile strength, which is 31.1% higher than that of the blank group. As evidently revealed by the stress–strain curve of the tensile test depicted in Figure 11b, the ultimate tensile stress and strain are the lowest for the blank group. As BF content increases, the stress or strain scales up noticeably, particularly in the test groups with a BF content of 1.0% and 1.5%. This indicates that BFs ameliorate the tensile strength and ductility of CPG.

### 3.2. Mixing BF and CW Test

#### 3.2.1. Flexural Strength

**Figure 12:**
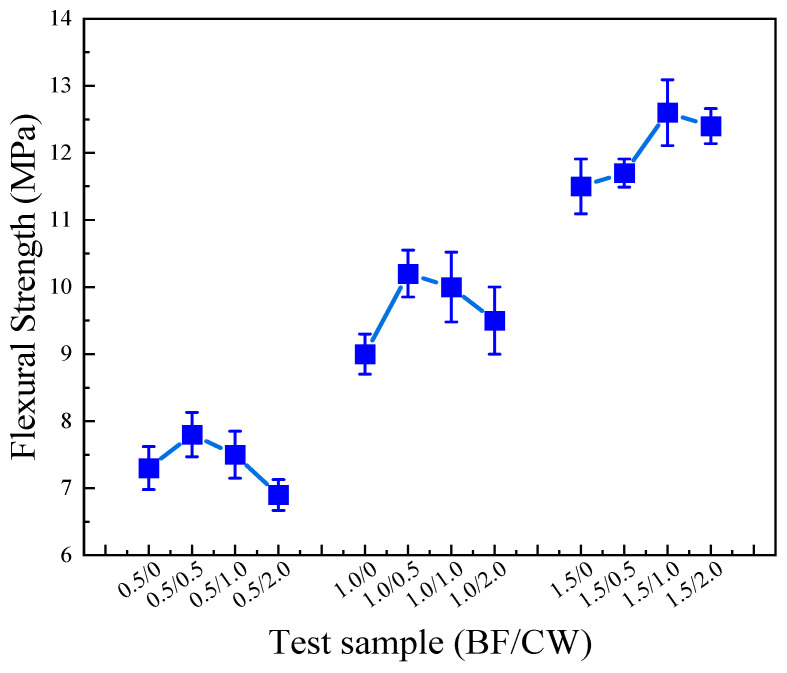
Flexural strength of gypsum samples mixed with 6 mm BF and CW (0.5/0.5 means that the content of 6 mm BF and CW is 0.5% and 0.5%, respectively, and the rest are similar).

**Figure 13:**
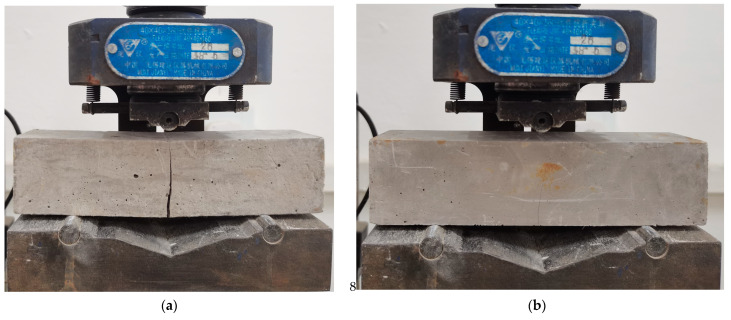
Failure pattern of sample (**a**): 1.5% BF; (**b**): 1.5% BF + 2.0% CW.

As illustrated by the flexural strength test results of the CPG mixed with 6 mm BF and CW in Figure 12, the flexural strength of CPG with 6 mm BF exhibits an initial augmentation and then a reduction as the content of CW is increased. In the test group with a fixed BF content of 0.5%, the CW content varied from 0 to 2.0%, which gives rise to flexural strengths of 7.3 MPa, 7.8 MPa, 7.5 MPa, and 6.9 MPa, respectively. Similarly, when the fixed BF dosage was 1.5%, the flexural strengths of each group were 11.5 MPa, 11.7 MPa, 12.6 MPa, and 12.4 MPa, separately. These findings suggest that cross-scale fiber blending can heighten the flexural strength of CPG, with an optimal ratio of BF to CW. The filling effect of whisker [38] ameliorates the compactness of the interface, bringing about increased frictional bond strength and ultimately higher flexural strength. Nevertheless, excessive whiskers tend to aggregate, which brings about increased porosity at the interface instead of densification [38]. Consequently, a higher whisker content brings about an abatement in flexural strength. Moreover, the stress–failure process revealed that the blended samples containing BF and CW exhibited narrower cracks, as depicted in Figure 13, indicating stronger cohesion.

#### 3.2.2. Compressive Strength

**Figure 14:**
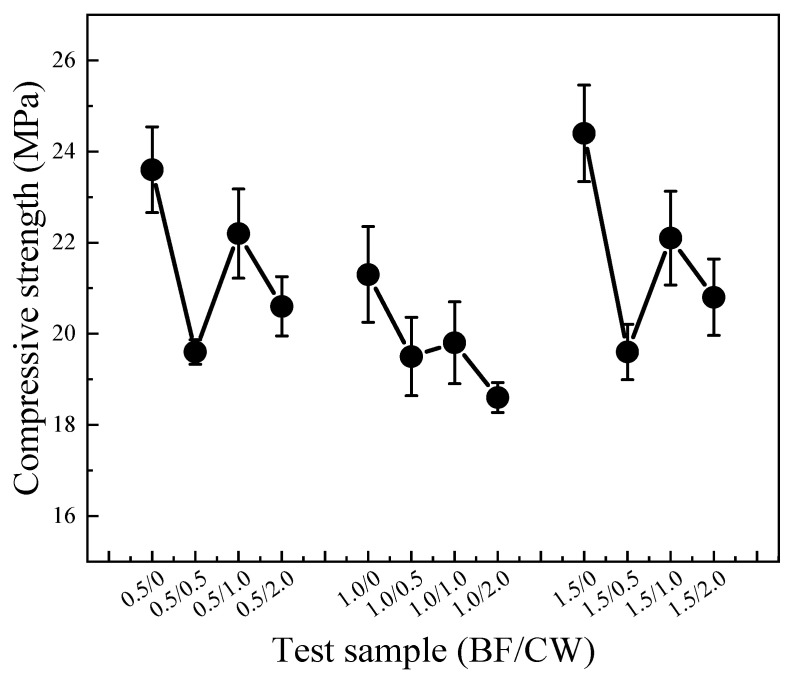
Compressive strength of gypsum samples mixed with 6 mm BF and CW.

Figure 14 exhibits the compressive strength test results of CPG samples mixed with 6 mm BF and CW. Unexpectedly, mixing BF and CW did not augment the compressive strength of the samples; instead, there was a decrease in strength. When the BF content is fixed at 0.5%, and 0, 0.5%, 1.0%, and 2.0% of CW are added, the corresponding compressive strengths are 23.6 MPa, 19.6 MPa, 22.2 MPa, and 20.6 MPa, separately. In the test group with an amount of 1.5%, the corresponding compressive strengths were 24.4 MPa, 19.6 MPa, 22.1 MPa, and 20.8 MPa, all of which exhibited a reduction in compressive strength. As evidently illustrated by the above experimental findings, when the content of CW is 0.5%, 1.0%, and 2.0%, the compressive strength initially increases and then decreases. Furthermore, when the content is 1.0%, the compressive strength is higher compared to the test groups mixed with 0.5% and 2.0%.

#### 3.2.3. Toughness

**Figure 15:**
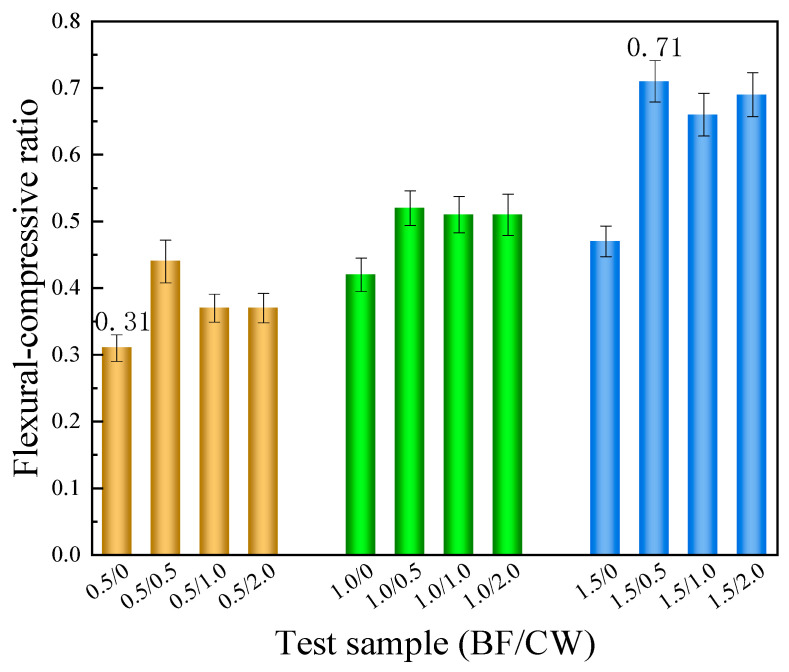
The flexural–compressive ratio of the samples mixing 6 mm BF and CW.

Based on the flexural and compressive strengths of the CPG samples blended with 6 mm BF and CW, the flexural–compressive ratio was calculated and presented in Figure 15. As a whole, the test groups incorporating both BF and CW exhibited higher flexural–compressive ratios compared to the group mixed with BF alone. The above test result signifies that the inclusion of cross−scale fibers positively influences the toughness of the CPG material. Apparently, the test group containing 1.5% BF content presented the highest flexural–compressive ratio. With the addition of 0.5%, 1.0%, and 2.0% CW, the ratios were elevated by 31.9%, 21.0%, and 27.7%, separately. It is noteworthy that the enhancement in toughness can be attributed to the presence of CW, which not only strengthens the flexural strength but also lowers the compressive strength.

#### 3.2.4. Water Absorption and Softening Coefficient

**Figure 16:**
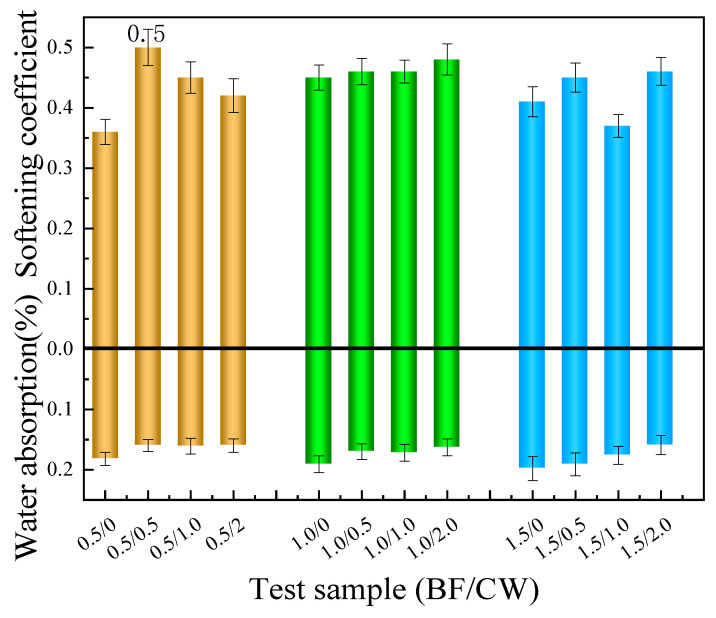
Water absorption and softening coefficient of samples mixed with 6 mm BF and CW.

Figure 16 displays the water absorption and softening coefficient of CPG blended with 6 mm BF and CW. As persuasively demonstrated by the analysis findings, the incorporation of CW in conjunction with BF yields a lowered water absorption rate and an elevated softening coefficient when compared to the sample comprising nothing but 6 mm BF. This phenomenon suggests that the addition of CW effectively compensates for the reduction in water resistance, resulting from the BF as a single dopant. Apparently, the group consisting of 0.5% 6 mm BF and 0.5% CW exhibited the highest softening coefficient, namely 0.50. As already pointed out by previous reports, nanoscale fibers possess the ability to effectively occupy void spaces [38], thereby impeding the creation of interconnected pores and consequently reinforcing the water resistance of the composite matrix.

#### 3.2.5. Tensile Strength

**Figure 17:**
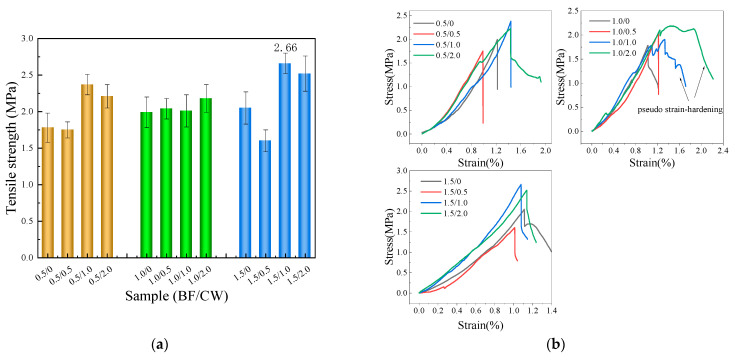
Tensile strength test results of samples mixed with 6 mm BF and CW (**a**): Average tensile strength; (**b**): typical stress–strain curve.

Figure 17a illustrates the tensile strength of CPG blended with 6 mm BF and CW. Except the 0.5/0.5 and 1.5/0.5 test groups, the tensile strength of the other test groups mixed with BF and CW was higher in comparison with the test group mixed with BF alone. In accordance with systematic and comprehensive analyses, the reduction in strength in certain groups may be attributed to the uneven dispersion of whiskers. Overall, the tensile strength of CPG was strikingly reinforced with the addition of 1.0% and 2.0% CW. Figure 17b represents the stress–strain curve, demonstrating that the use of 1.0% or 2.0% CW with 6 mm BF is conducive to augmenting the ultimate tensile stress and strain of CPG. It is extremely noteworthy that, in the 1.0/1.0 and 1.0/2.0 test groups, a pseudo-strain strengthening effect was observed during the stretching process, with the maximum tensile strain exceeding 2%. On the basis of the above exploration, we can arrive at the conclusion that CW possesses the ability to inhibit the initiation and propagation of micro-cracks, scale up the crack initiation strength of the matrix, and may give rise to the formation of multiple micro-cracks during tension. Nonetheless, the development of these micro-cracks into macro-cracks becomes slower and more challenging [30,31], which ultimately results in the optimization of tensile strength and ductility in the matrix.

### 3.3. Mechanism Analysis

#### 3.3.1. Monofilament Number and Interface Area Calculation

During the test process, when adding nothing more than BFs, it was discovered that a 6 mm BF exerted the most conspicuous influence on elevating the flexural, compressive, and tensile strength. Furthermore, as the dosage increased, there was an upward trend in the mechanical properties. For this reason, the reinforcement effect of the mechanical properties is bound up with multiple factors, such as the quantity of monofilament fibers in CPG, as well as the interface area between the fibers and the matrix. The estimation of the number and interfacial area of monofilament fibers can be determined using Formulas (1) and (2) [35]:

Q = (1000C/ρ)/[π × (0.5A/1000)2 × B](1)

S = (A/1000) × πBQ(2)

where: Q stands for the number of monofilament fibers; S represents the interfacial area, mm^2^; the total mass of dry matter is set to 1000 g; ρ is the fiber density, 2.65 g/cm^3^; A stands for the fiber diameter, µm; B represents the fiber length, mm; C stands for the fiber mass fraction, %. The calculation results are listed in Table 3.

In accordance with the calculation results presented in Table 3, while maintaining a constant fiber content, the number of individual fibers progressively lowers as the fiber length increases, while the interfacial area remains unchanged. Conversely, with constant fiber length, both the number of individual fibers and the interfacial area increase as the fiber dosage augments. More importantly, the interfacial area exhibits periodic variations in conjunction with alterations in mass fraction and fiber length. For instance, at a content of 1.5%, the number of individual fibers for 3 mm, 6 mm, 9 mm, and 18 mm BFs were determined to be 14,222.2, 7111.1, 4740.7, and 2370.4, separately. While the number of individual fibers gradually decreases, the corresponding interfacial area measures 1741.7 mm^2^. Notwithstanding the fact that the 3 mm BF exhibits the highest number of individual fibers, these shorter fibers are susceptible to being dislodged and losing their effectiveness. On the contrary, longer fibers can impart greater friction and resistance during the peeling process; nevertheless, the number of individual fibers in the 9 mm and 18 mm BFs is lower, triggering uneven dispersion. To go further, these longer fibers tend to aggregate, thus posing challenges with regard to how we can effectively materialize uniform dispersion during the stirring process. In contrast, the 6 mm fibers yield the most favorable results with respect to reinforcing mechanical properties. Upon incorporating 0.5%, 1.0%, and 1.5% of 6 mm BFs into the matrix, a substantial increase is observed in both the number of individual fibers and the interfacial area. This augmentation significantly optimizes the cohesion among matrix components, ultimately advancing the enhancement of mechanical properties.

#### 3.3.2. SEM Analysis

**Figure 18:**
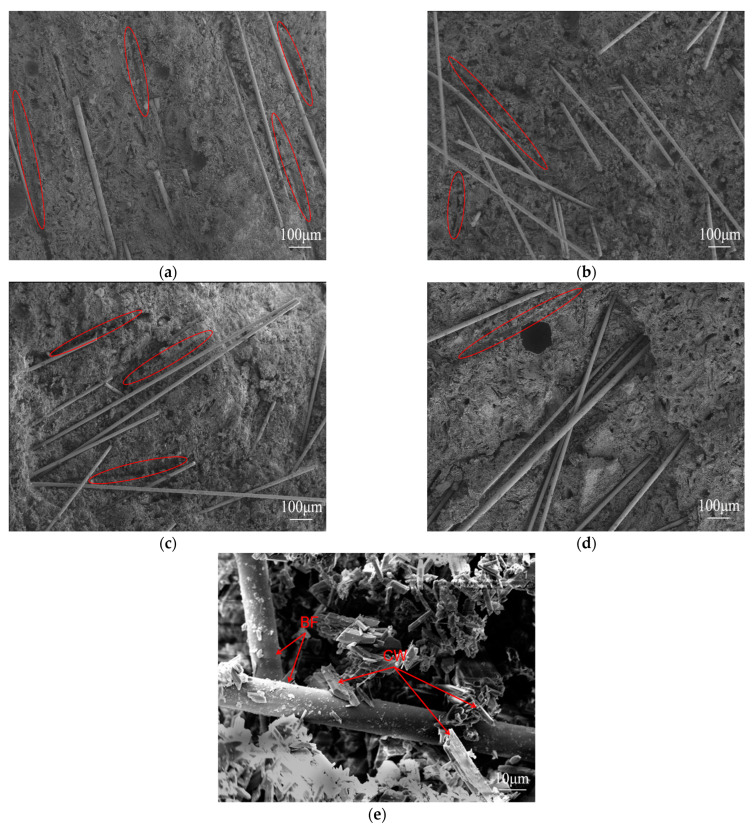
SEM image of fracture surface of tensile sample (the red markings show the traces left behind after the fibers were pulled out). (**a**) 1.5% 3 mm BF; (**b**) 1.5% 6 mm BF; (**c**) 1.5% 9 mm BF; (**d**) 1.5% 18 mm BF; (**e**) the binding state of BF and the matrix.

The fracture surface of the tensile sample with 1.5% BF content was examined using SEM testing. Figure 18a–d represent samples containing 3 mm, 6 mm, 9 mm, and 18 mm of BFs, separately. As evidently displayed in the above figures, the dispersion of 3 mm, 9 mm, and 18 mm BFs in CPG exhibited a certain degree of orientation and aggregation, suggesting less satisfactory dispersion uniformity. For another, the 6 mm BF displayed a more uniform and chaotic dispersion pattern, which contributes to the optimization of mechanical properties. Nonetheless, the combination of the fibers and the matrix does not appear to be dense, which results in the fibers being mostly pulled out during the stress process. This is supported by the presence of remaining fiber traces in Figure 18a–d. Figure 18e is the SEM image of the sample mixed with 1.5% BF and 2.0% CW. This image reveals an interlacing of CW and BF, with CW embedded within the gypsum crystal. This interlacing mechanism serves to mitigate stress concentration and retard stress generation. Moreover, the introduction of whiskers is advantageous for averting crack propagation, facilitating crack deflection, and thereby elongating crack propagation pathways while dissipating energy. In this regard, whiskers play a pivotal role in optimizing the mechanical properties of the material under investigation [30].

#### 3.3.3. MIP Analysis

**Figure 19:**
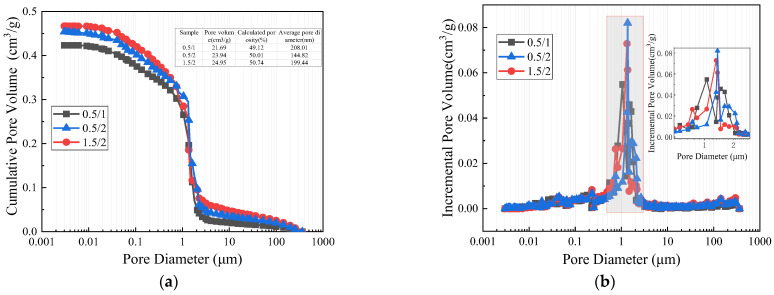
MIP test result (**a**): Cumulative pore volume; (**b**): incremental pore volume.

Samples from groups 0.5/1, 0.5/2, and 1.5/2 were subjected to MIP testing, and the results are presented in Figure 19. The sample containing 0.5% 6 mm BF and 1.0% CW exhibited the lowest porosity, followed by the 0.5/2 and 1.5/2 samples. As compared with the 0.5/1 and 0.5/2 groups, the 0.5/2 group with a higher CW content exhibited higher pore volume and porosity than the 0.5/1 group. Nonetheless, the average pore diameter was significantly lessened, indicating that the addition of CW contributed to pore refinement. This refinement is beneficial in lowering the macroscopic defects of CPG, thereby elevating its flexural strength, softening coefficient, and tensile strength. In comparison with the 0.5/2 and 1.5/2 groups, the 1.5/2 group with a higher BF content displayed higher pore volume and porosity than the 0.5/2 group. This increase in porosity is ascribed to the presence of pores at the weaker bonding interface between BF and the matrix. In some sense, a higher BF content results in weaker bonding interfaces. This analysis result is enlightening on the phenomenon of increased water absorption rate in CPG after the incorporation of BF. In comparison with the 1.5/2 and 0.5/1 groups, it is evident that augmenting the amount of BF and CW simultaneously increases pore volume and porosity in a particularly conspicuous manner, which is the leading cause for the reduction in the compressive strength of CPG after mixing BFs and CW.

## 4. Conclusions

The effects of basalt fiber and calcium carbonate whisker on the properties of CPG were studied systematically on the basis of individual and mixed tests. The main conclusions are displayed as follows:

The inclusion of BF tremendously elevates the strength of CPG, thereby ameliorating toughness, the flexural–compressive ratio, tensile strength, and strain capacity. Nevertheless, it can also increase water absorption, consequently lessening water resistance. Apparently, adding 6 mm BF can bring about the most substantial impact, particularly at 1.5% content, where it increases flexural and compressive strengths by 92.2% and 32.0%, respectively, in comparison with the control group.

The concurrent addition of 6 mm BF and CW to CPG immensely reinforces its flexural strength and toughness, albeit at the expense of compressive strength. Moreover, the inclusion of CW alongside BF ameliorates the softening coefficient and water resistance of CPG, which clearly suggests that CW effectively compensates for the diminished water resistance resulting from BF incorporation alone. Furthermore, CW dramatically contributes to the augmentation of tensile strength and strain capacity in CPG. Apparently, the inclusion of 1.0% 6 mm BF and 2.0% CW in CPG reveals a notable pseudo-strain strengthening effect during the tensile process, with a remarkable maximum tensile strain exceeding 2%.

As already demonstrated by the microscopic tests, the dispersion uniformity of 6 mm BF in CPG surpasses that of fibers with other lengths. Nevertheless, the bonding between the fiber and matrix is not adequate as anticipated, resulting in the fiber being predominantly pulled out when subjected to stress. MIP tests have indicated that augmenting the quantity of basalt fiber results in an elevation in pore volume and porosity of CPG. Likewise, augmenting the amount of CW also elevates porosity, but it contributes to refining the pores and enhancing the cohesion of the matrix. Hence, this betterment in pore structure reinforces the flexural strength, softening coefficient, and tensile strength of CPG.

This study provides a new idea for improving the mechanical properties of phosphogypsum and provides a scientific basis for promoting the application of industrial by-product gypsum in the field of building materials.

## Figures and Tables

**Table 1:** Performance parameters of BFs and CW.

Materials	Diameter (μm)	Length (μm)	Density (g/cm^3^)	Tensile Strength (MPa)
BFs	13	3, 6, 9, 18	2.65	3800–4800
CW	1–2	20–30	2.80	−

**Table 2:** Mix proportion.

Experimental Group	CPG	Water	Retarder	BFs		CW (wt%)
				Length (mm)	Dosages (wt%)	
Individual tests: BFs	1	0.45	0.001	3	0/0.5/1.0/1.5	−
	1	0.45	0.001	6	0/0.5/1.0/1.5	−
	1	0.45	0.001	9	0/0.5/1.0/1.5	−
	1	0.45	0.001	18	0/0.5/1.0/1.5	−
Mixed tests:BFs + CW	1	0.45	0.001	6	0.5	0.5/1.0/2.0
	1	0.45	0.001	6	1.0	0.5/1.0/2.0
	1	0.45	0.001	6	1.5	0.5/1.0/2.0

**Table 3:** Calculation results of the number of monofilament fibers and the interfacial area.

Mass Fraction of Fibers (C, %)	Fiber Density (ρ, g/cm^3^)	Fiber Diameter (A, μm)	Fiber Length (B, mm)	Monofilament Quantity (Q)	Interfacial Area (S, mm^2^)
0.5	2.65	13	3	4740.7	580.6
1.0	2.65	13	3	9481.5	1161.1
1.5	2.65	13	3	14,222.2	1741.7
0.5	2.65	13	6	2370.4	580.6
1.0	2.65	13	6	4740.7	1161.1
1.5	2.65	13	6	7111.1	1741.7
0.5	2.65	13	9	1580.2	580.6
1.0	2.65	13	9	3160.5	1161.1
1.5	2.65	13	9	4740.7	1741.7
0.5	2.65	13	18	790.1	580.6
1.0	2.65	13	18	1580.2	1161.1
1.5	2.65	13	18	2370.4	1741.7

## Data Availability

Dataset available on request from the authors.
